# Preclinical systolic dysfunction relating to ankle-brachial index among high-risk PAD population with preserved left ventricular ejection fraction

**DOI:** 10.1038/s41598-024-52375-y

**Published:** 2024-03-14

**Authors:** Yueh-Hung Lin, Kuo-Tzu Sung, Cheng-Ting Tsai, Yau-Huei Lai, Chi-In Lo, Fa-Chang Yu, Wei-Ran Lan, Ta-Chuan Hung, Jen-Yuan Kuo, Charles Jia-Yin Hou, Chih-Hsuan Yen, Ming-Cheng Peng, Hung-I. Yeh, Ming-Ting Wu, Chung-Lieh Hung

**Affiliations:** 1 Department of Medicine, Mackay Medical College, New Taipei City, 25245 Taiwan; 2 Division of Cardiology, Departments of Internal Medicine, Mackay Memorial Hospital, No. 92, Sec. 2, Zhongshan N. Road, Taipei, 10449 Taiwan; 3 School of Medicine, Institute of Clinical Medicine, National Yang-Ming University, Taipei, Taiwan; 4 Mackay Junior College of Medicine, Nursing, and Management, Taipei City, 11260 Taiwan; 5 Division of Cardiology, Department of Internal Medicine, Hsinchu MacKay Memorial Hospital, Hsin-chu City, 30071 Taiwan; 6 Department of Radiology, Kaohsiung Veterans General Hospital, No. 386, Dazhong 1St Road, Kao-hsiung City, 81362 Taiwan; 7 Faculty of Medicine, School of Medicine, National Yang Ming Chiao Tung University, Taipei, 11221 Taiwan

**Keywords:** Cardiology, Medical research, Risk factors

## Abstract

Peripheral artery disease (PAD) shares common clinical risk factors, for example, endothelial dysfunction, with preserved ejection fraction (LVEF) heart failure (HFpEF). Whether PAD is associated with preclinical systolic dysfunction and higher HF risk among individuals presenting preserved LVEF remains uncertain. We retrospectively included outpatients with at least one known or established cardiovascular (CV) risk factor with LVEF ≥ 50%. Patients were categorized into high risk and low risk of developing PAD (PAD vs Non-PAD) by ankle-brachial index (ABI) (≤ 0.90 or > 1.4) and further stratified based on their history of HFpEF (HFpEF vs. Non-HFpEF), resulting in the formation of four distinct strata. Preclinical systolic dysfunction was defined using dedicated speckle-tracking algorithm. A total of 2130 consecutive patients were enrolled in the study, with a median follow-up of 4.4 years. The analysis revealed a higher prevalence of high risk of developing PAD in patients with HFpEF compared to those without HFpEF (25.1% vs. 9.4%). Both high risk of developing PAD and HFpEF were independently associated with preclinical systolic dysfunction (global longitudinal strain, GLS ≥ − 18%) (odds ratio, OR: 1.38; 95% confidence interval, CI: 1.03–1.86). In comparison to patients at low risk of developing PAD without HFpEF (Non-PAD/Non-HFpEF group), those categorized as having a high risk of developing PAD with HFpEF (PAD/HFpEF group) exhibited the most impaired GLS and a heightened susceptibility to heart failure hospitalization (hazard ratio, HR: 6.51; 95% CI: 4.43–9.55), a twofold increased risk of all-cause mortality (HR: 2.01; 95% CI: 1.17–3.38), cardiovascular mortality (HR: 2.44; 95% CI: 1.08–5.51), and non-cardiovascular mortality (HR: 1.78; 95% CI: 0.82–3.84). A high risk of developing PAD was strongly linked to impaired preclinical systolic function and an increased likelihood for subsequent hospitalization for HF, all-cause mortality, CV mortality and non-CV mortality. There is a clear need for preventive strategies aimed at reducing hospitalizations for HF and mortality in this high-risk population.

## Introduction

Heart failure (HF) with preserved ejection fraction (HFpEF) remains a public health problem associated with high morbidity and mortality burden^1^. While it has been proposed that macrovascular disease (e.g., coronary artery disease [CAD]) is a common comorbidity of HFpEF; on the other hand, accumulating data have shown microvascular disease as potential pathophysiology of HFpEF^2,3^. Peripheral artery disease (PAD), a well-known predictor of CAD events and mortality, has been proposed to arise from endothelial dysfunction (ED) or inflammation, leading to subsequent end-organ ischemia and the development of atherosclerosis^4^. While patients with PAD frequently share common risk factors with CAD, those with known PAD further display a higher risk for all relevant cardiovascular events or all-cause mortality compared to those without PAD among individuals free of known cardiovascular (CVD) in the MESA study^5^. Additionally, patients with acute decompensated heart failure (ADHF) and concomitant PAD showed higher likelihood of readmission^6,7^. As HFpEF and PAD share many similar cardiovascular risk factors and frequently coexist within the same patient population^8^, thus, it is not surprising that PAD is associated with an increased risk of mortality in patients with HFpEF^6,9^.

To date, the ankle-brachial index (ABI) is a clinically feasible, convenient, and non-invasive tool for documenting the presence of lower-extremity PAD, and has been widely used in clinical settings. Therefore, this study aimed to investigate whether subjects with high PAD risk assessed by ABI test in a large-scale outpatient population may share similar pathophysiology of HFpEF by manifesting impaired preclinical systolic dysfunction.

## Methods

### Study subjects and design

A total of 2130 consecutive patients with PAD risk who presented at the cardiovascular outpatient clinics at Mackay Memorial Hospital from August 2009 to Dec 2014 were recruited retrospectively. Eligible study participants had at least one known or established cardiovascular (CV) risk factor including senescence (male > 45 years, female > 55 years), history of hypertension, type 2 diabetes, hypercholesterolemia, high-density lipoprotein cholesterol (male < 40 mg/dl, female < 50 mg/dl), known heart failure (HF), cerebrovascular event, CAD, or smoking history.

Patients with documented significant valvular heart disease (more than moderate valvular heart disease), whether or not they had undergone surgical correction, documented reduced LV systolic HF (left ventricular ejection fraction ≤ 40%), congenital heart disease, recent acute coronary syndrome and known cardiomyopathy were excluded from the data collection process at the time when patient information was gathered. Demographic data and medical history were all collected by three independent cardiologists during face-to-face interviews. All participants underwent biochemical examination.

As patients with specific risk factors such as aging, DM, HTN, and smoking were at a higher risk for PAD, we initiated a screening process for PAD. All patients underwent assessments that included measurements of right and left brachial-ankle pulse wave velocity (ba-PWV), femoral-ankle PWV (fa-PWV), right ABI and left ABI. Transthoracic echocardiography was performed within two weeks of ABI study to exclude structural abnormalities and to assess preclinical systolic dysfunction using speckle-tracking based deformational measures. The diagnosis of HFpEF was established within a period of 3 months from the ABI study.

Initially, patients were segregated into two groups based on their risk of developing PAD: one group designated as the PAD group with a high risk, and the other as the Non-PAD group with a low risk, determined by the results of the ABI study. Subsequently, within these groups, further categorization was performed based on their risks of developing PAD with or without HFpEF. This classification process is visually represented in Supplement Fig. 1, serving as the foundational framework for our subsequent analysis of hospitalization rates for heart failure, all-cause mortality, CV mortality, and non-CV mortality. The study was approved by the local Institutional Review Board (MacKay Memorial Hospital Institutional Review Board Committee) (15MMHIS031e) and informed consent was waived due to retrospective study nature. The conduction of this study complied with the Declaration of Helsinki.

### Measurement of anthropometrics and baseline risk factors

Anthropometric parameters, including height, body weight, and waist circumference, were measured by experienced study nurses. Hypertension was defined as a systolic blood pressure (SBP) ≥ 140 mmHg and/or diastolic blood pressure (DBP) ≥ 90 mmHg from two different measures or the use of antihypertensive agents. Hypercholesterolemia was defined as total cholesterol ≥ 200 mg/dl, low-density lipoprotein cholesterol ≥ 130 mg/dl, or the use of lipid-lowering medications (statins or fibrates). Diabetes was defined as a fasting blood glucose level > 126 mg/dl or use of DM medication, while smoking history was defined as being an ex-smoker or current tobacco use.

CAD was defined as a condition characterized by either a documented history of acute coronary syndrome, clinical symptoms indicative of CAD, or the presence of coronary artery stenosis exceeding 50%, as determined through CT or angiography. This definition encompasses cases both with and without percutaneous intervention (such as angioplasty) or those requiring coronary artery bypass grafting. The diagnosis of CAD was confirmed at the time when patient information was gathered.

Renal function was evaluated by estimated glomerular filtration rate as: eGFR = 186.3 × (serum creatinine^−1.154^) × (age^−0.203^) × 0.742 (if female).

### Measurement of pulse wave velocity (PWV) or ankle-brachial index (ABI)

**Figure 1:**
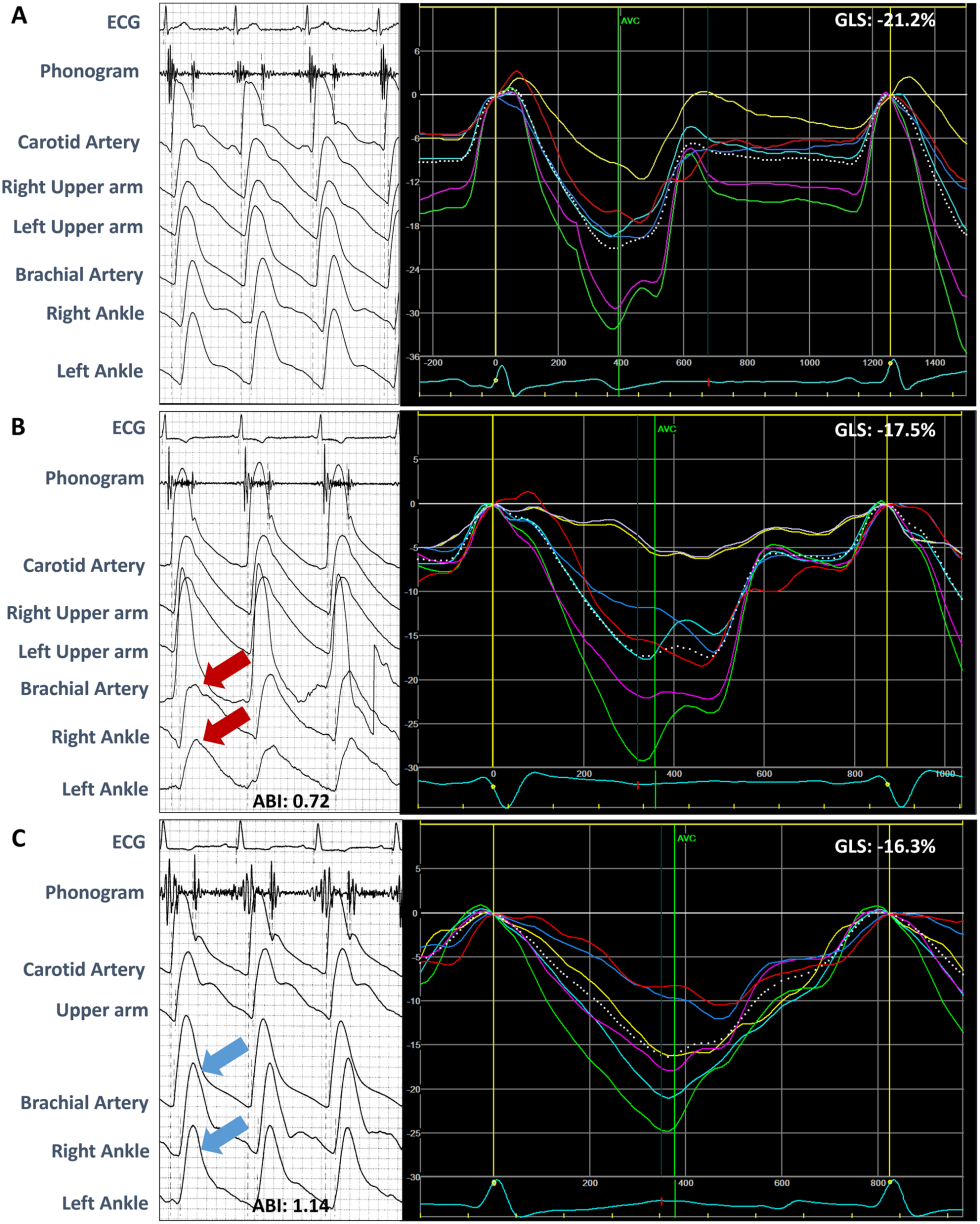
Illustration for ABI/PWV waveforms and global longitudinal systolic function by deformation measure. (**A**) One patient with normal ABI value (1.01) yet without clinical HF diagnosis (as Non-PAD/Non-HFpEF) with relatively preserved longitudinal systolic strain (right, from 4-chamber view); (**B**) another patient in this study with normal ABI value (1.14) with prior HFpEF diagnosis (as Non-PAD/HFpEF), with normal arterial tracing waveforms from lower extremities (left, blue arrows) and globally diminished longitudinal systolic strain pattern (right, from 4-chamber view). ECG, peripheral artery disease; HEpEF, heart failure with preserved ejection fraction; GLS, global longitudinal strain; LVEF, left ventricular ejection fraction.

The ABI and PWV were measured by an experienced technician a single rater. After resting in the supine position for 5 min, bilateral ba-PWV, fa-PWV, systolic blood pressure, and diastolic blood pressure from the four limbs were measured using an automated machine (VP-2000; Collin Corp., Japan) gated with electrocardiogram (ECG) (Fig. 1). PWV as one arterial stiffness measure was calculated as the distance between the two arterial sites divided by the time delay between the two arterial point sites and presented as centimeters per second. The right and left ABI were calculated by the highest pressure on the dorsal or posterior tibial artery on the right and left sides, respectively, and by the highest brachial pressure on either side. Of the two ABI measurements for each patient, we selected the lowest ABI for study use.

### The establishment of high risk of developing PAD individuals

According to the recommendations of the American College of Cardiology and the American Heart Association (ACC/AHA) guidelines, ABI results should be reported consistently as follows: non-compressible values defined as greater than 1.40, normal values falling within the range of 1.00–1.40, borderline values within 0.91–0.99, and abnormal values at 0.90 or less. In our study, we followed the recommendation for defining high risks of developing peripheral artery disease (PAD) by considering participants with an ankle-brachial index (ABI) ≤ 0.90 or ABI > 1.4 on either side of the leg. It’s essential to emphasize that these participants were not subjected to imaging studies to confirm the presence of PAD, and they did not display symptoms of PAD. Consequently, we opted to refer to them as having “high risk of developing PAD” rather than explicitly labeling them as having PAD.

### Measurement of echocardiography parameters

Two-dimensional echocardiography was performed according to the American Society of Echocardiography recommendations. Left ventricular ejection fraction (LVEF) was measured using Simpson biplane method^10^ and the left ventricular (LV) mass index, relative wall thickness (RWT), left arterial (LA) diameter, isovolumic relaxation time (IVRT), and deceleration time (DT) were also measured. In the septum and lateral annulus, LV e′ was measured using tissue Doppler, and the E/e′ ratio was calculated. Advanced echocardiography imaging using strain imaging to evaluate cardiac function was performed along with the measurement of global longitudinal strain (GLS) (Fig. 1) and global circumferential strain (GCS). The offline workstation, algorithm used and variations for speckle-tracking of measures of GLS/GCS from our laboratory were published previously^10^. Preclinical systolic dysfunction was defined as impaired GLS with a value of GLS ≥ − 18%)^11^.

### Laboratory measurements

Overnight fasting blood serum and plasma samples were collected for glucose, lipid profiles (total cholesterol, triglyceride, low-density lipoprotein cholesterol, and high-density lipoprotein cholesterol), and biochemical measurements, including renal function. Serum samples were collected in standard sampling tubes or tubes containing separate gels. After ensuring individualized patient samples, calibrators and controls were set at ambient temperature (20–25 °C), and the measurements were taken within 2 h. High-sensitivity C-reactive protein (hs-CRP) levels were determined using a highly sensitive latex particle-enhanced immunoassay using Elecsys 2010 (Hitachi Corp. Hitachinaka Ibaraki, Japan). Serum B type natriuretic peptide (BNP) concentrations were measured using fluorescence immunoassay microtiter plate with a coefficient of variation (CV) 10.4% (Alere Biosite Triage, San Diego Inc. Ca, USA). Renal function was determined by estimating glomerular filtration rate (eGFR), calculated using the Modification of Diet in Renal Disease (MDRD) formula.

### Statistical analysis

Continuous data is represented as the mean along with its standard deviation (SD), while categorical variables are presented as proportions or percentages. To examine the trends in demographic information based on the risk of developing PAD and HFpEF, the Mann–Whitney U test was employed. In order to explore the relationships between ABI parameters (including minimal ABI, fa-PWV, and ba-PWV) and cardiac functional indices such as E/e′, GLS, and GCS, univariate linear regression models were utilized. These models allowed us to assess the associations between these variables. We employed a series of statistical models, both univariate and multivariate, adjusting for potential confounding factors. These models were applied to four strata based on the high risk of developing PAD and HFpEF, using the Non-PAD/Non-HFpEF group as the reference.

In the first model, we adjusted for age and sex, recognizing the influence of these demographic factors on the outcomes. Subsequently, the second model included additional adjustments for age, sex, and body mass index (BMI), taking into account the potential impact of BMI on the results. The third model incorporated a more extensive set of adjustments, considering age, sex, as well as hypertension, diabetes, coronary CAD, atrial fibrillation, hyperlipidemia, smoking status, eGFR and LV mass index.

Results from these uni- and multivariate Cox models were analyzed for various endpoints, including hospitalization for HF), all-cause mortality, CV mortality, and non-CV mortality. To visualize the differences in these outcomes among groups with varying risks of developing PAD and HFpEF, we used the Kaplan–Meier survival estimator to generate survival curves and make comparisons between the different risk groups.

All statistical analyses were two-tailed, and *P* < 0.05 was considered statistically significant. Statistical analyses were performed using IBM Statistics (version 26.0; SPSS Inc., Armonk, NY, USA).

## Results

### Baseline characteristics

Throughout the study duration, a total of 2130 patients were included. Table 1 presents a comprehensive overview of the baseline clinical characteristics of the study population, delineating individuals at both high and low risks of developing PAD. The median following time was 4.4 years (interquartile range: 1.6–7.3 years) with a mean age of 62.2 years. Among the total patient population, 252 patients (12%) were diagnosed as having a high risk of developing PAD. The majority of these participants were asymptomatic. These patients exhibited several distinct characteristics when compared to those at low risk of developing PAD, including a higher likelihood of being male, older and have higher systolic blood pressure. Additionally, they had higher levels of fasting glucose level, HbA1c, BNP, hs-CRP, and a lower total cholesterol, low-density cholesterol, and eGFR as detailed in Supplemental Table 1.

**Table 1:** Baseline characteristics.

	All (n = 2,130)	Non-PAD (n = 1,878)	PAD (n = 252)	P value
Baseline characteristics				
Age (years)	62.20 ± 12.80	61.24 ± 12.34	69.36 ± 13.90	< 0.001
Sex, female (%)	1125 (52.8%)	1003 (53.4%)	112 (48.4%)	0.136
BMI (kg/m^2^)	25.80 ± 4.35	25.81 ± 4.35	25.63 ± 4.36	0.612
Systolic blood pressure (mmHg)	136.60 ± 21.33	135.31 ± 20.64	146.30 ± 23.86	< 0.001
Diastolic blood pressure (mmHg)	78.54 ± 13.19	78.50 ± 13.00	78.83 ± 14.51	0.519
Waist (cm)	88.74 ± 11.18	88.50 ± 11.10	90.50 ± 11.66	0.014
Medical history				
Active smoker, n (%)	499 (23.4%)	427 (22.7%)	72 (28.6%)	0.04
Hypertension, n (%)	1630 (76.5%)	1443 (76.8%)	187 (74.2%)	0.355
Diabetes, n (%)	722 (33.9%)	593 (31.6%)	129 (51.2%)	< 0.001
Atrial fibrillation, n (%)	108 (5.1%)	75 (4.0%)	33 (13.6%)	< 0.001
Heart failure, n (%)	327 (15.4%)	245 (13%)	82 (32.5%)	< 0.001
Coronary artery disease, n (%)	380 (17.8%)	317 (16.9%)	63 (25.1%)	0.001
Prior MI	80 (3.8%)	68 (3.6%)	12 (4.8%)	0.371
Dyslipidemia (%)	1162 (54.6%)	1044 (55.6%)	118 (46.8%)	0.009
Medications used				
ACEi/ARB, n (%)	1118 (52.5%)	970 (51.7%)	148 (58.7%)	0.035
Beta blocker, n (%)	1162 (54.6%)	1023 (54.5%)	139 (55.4%)	0.787
Statin, n (%)	622 (29.2%)	549 (29.2%)	73 (29.1%)	0.961
Antiplatelet, n (%)	619 (26.9%)	507 (26.9%)	112 (45.2%)	< 0.001

BMI, body mass index; LDL, low density cholesterol; eGFR, estimated glomerular filtration rate; Hs-CRP, high-sensitivity C-reactive protein; BNP, B-type natriuretic peptide; ACEi, angiotensin-converting enzyme inhibitors; ARB, angiotensin II receptor blockers.

Furthermore, within the high risk of developing PAD group, there was a higher prevalence of smoking (28.6% vs. 22.7%) and diabetes mellitus (51.2% vs. 31.6%) compared to the low-risk group. Atrial fibrillation (13.6% vs. 4%), CAD (25.1% vs. 16.9%), and history of stroke (6% vs. 2.1%) were also more common among those at high risk for developing PAD group, while the prevalence of dyslipidemia was lower (46.8% vs. 55.6%). Both groups had a known history of HFpEF, but it was more prevalent in the high risk of developing PAD group than in the low risk of developing PAD group (32.5% vs. 13%).

In terms of medication usage, in the high risk of developing PAD group, there was a higher utilization of ACE inhibitors/ARBs (58.7% compared to 51.7%) and antiplatelet medications (45.2% compared to 26.9%) when compared to the low risk of developing PAD group. However, the use of beta-blockers (55.4% compared to 54.5%) and statins (29.1% compared to 29.2%) was similar between the two groups.

### The associations of PAD in relation to vascular measures and cardiac function

We further categorized the patients into four groups based on their risks of developing PAD and HFpEF. Table 2 presents the cardiac function assessed through echocardiography in these groups. When compared to the reference group comprising individuals with low risk of developing PAD and without HFpEF (Non-PAD/Non-HFpEF), patients diagnosed with a high risk of developing PAD but not HFpEF (PAD/Non-HFpEF) exhibited impaired diastolic function. This was evident from their lower septal e′ velocity (5.97 cm/s versus 6.49 cm/s) and higher E/e′ ratio (11.07 versus 9.92). Additionally, these patients displayed impaired GLS (− 18% versus − 19%).

**Table 2:** The Associations among PAD, HFpEF and Cardiac Structure/Function.

	All (n = 2130)	Non-PAD, Non-HFpEF (n = 1633)	PAD, Non-HFpEF (n = 170)	Non-PAD, HFpEF (n = 245)	PAD, HFpEF (n = 82)	P value
LV mass index (g/m^2^)	85.86 ± 22.64	83.82 ± 21.27	87.57 ± 23.79	94.55 ± 26.77*^†^	94.93 ± 23.82*	< 0.001
Relative wall thickness	0.43 ± 0.074	0.43 ± 0.073	0.44 ± 0.073	0.44 ± 0.077*	0.46 ± 0.081*	< 0.001
LA diameter (mm)	33.14 ± 6.23	32.64 ± 6.18	33.32 ± 6.25	35.23 ± 5.29*^†^	36.57 ± 7.50*^†^	< 0.001
LVEF (%)	64.71 ± 6.63	65.09 ± 6.51	64.23 ± 6.64	62.99 ± 6.70*	63.11 ± 7.79*	< 0.001
IVRT (ms)	85.35 ± 22.69	85.10 ± 22.81	84.23 ± 23.75	88.33 ± 20.82	83.48 ± 22.87	0.147
DT (ms)	226.18 ± 64.57	223.84 ± 57.30	228.84 ± 67.16	238.10 ± 89.04*	231.34 ± 97.33	0.010
Septal e′ (cm/s)	6.27 ± 2.08	6.49 ± 2.05	5.97 ± 2.25*	5.43 ± 1.77*	5.16 ± 2.00*^†^	< 0.001
Lateral e′ (cm/s)	8.02 ± 2.71	8.25 ± 2.67	7.73 ± 2.93	7.08 ± 2.56*	6.83 ± 2.61*	< 0.001
E/e′	10.59 ± 4.82	9.92 ± 3.83	11.07 ± 5.10*	13.07 ± 7.28*^†^	15.81 ± 6.83*^†‡^	< 0.001
GLS (%) (n = 2106)	− 18.71 ± 2.97	− 19.07 ± 2.74	− 18.31 ± 2.98*	− 17.53 ± 3.44*^†^	− 16.03 ± 3.40*^†‡^	< 0.001
GCS (%) (n = 2022)	− 20.15 ± 4.86	− 20.38 ± 4.80	− 19.75 ± 5.30	− 19.43 ± 4.63*	− 18.27 ± 5.32*	< 0.001

LV mass index, left ventricle mass index; LA diameter, left atrial diameter; LVEF, left ventricular ejection fraction; IVRT, isovolumetric relaxation time; DT, deceleration time; GLS, global longitudinal strain; GCS, global circumferential strain.

*P < 0.05 compared to Group 1, ^†^P < 0.05 compared to Group 2, ^‡^P < 0.05 compared to Group 3.

Patients with a high risk of developing PAD and HFpEF (PAD/HFpEF group) showed several differences compared to the Non-PAD/Non-HFpEF reference group: they had a higher left ventricular (LV) mass index (94.93 g/m^2^ versus 83.82 g/m^2^), higher relative wall thickness (0.46 versus 0.43), a larger left atrium diameter (36.57 mm versus 32.64 mm), and a lower left ventricular ejection fraction (63% versus 65%). In terms of tissue Doppler imaging parameters, patients with high risk of developing PAD and HFpEF exhibited lower septal e′ velocity (5.16 cm/s versus 6.49 cm/s), lower lateral e′ velocity (6.83 cm/s versus 8.25 cm/s) and increased E/e′ (15.81 versus 9.92) compared with Non-PAD/Non-HFpEF reference group. Additionally, analysis using speckle-tracking echocardiography revealed that patients with high risk of developing PAD and HFpEF had lower global longitudinal strain (− 16% versus − 19%) and lower circumferential strain (− 18% versus − 20%), compared with Non-PAD/Non-HFpEF reference group.

Table 3 displays the results of the linear regression analysis, examining the relationship between ABI/PWV parameters and indicators of cardiac functional performance. The findings reveal that more unfavorable vascular measures, characterized by lower ABI values, are significantly associated with deteriorated E/e′ (β-Coef, − 6.52; 95% CI, − 8.05 to − 5.00, *P* < 0.05), GLS (β-Coef, − 2.56; 95% CI, − 3.53 to − 1.6,* P* < 0.05), and GCS (β-Coef, − 2.04; 95% CI, − 3.66 to − 0.42, *P* < 0.05.

**Table 3:** ABI parameter vs cardiac functional indices.

Variables	Beta coefficient	95% confidence interval		P value
		Lower bound	Upper bound	
E/e′				
ABI (min)	− 6.52	− 8.05	− 5.00	< 0.001
fa-PWV (max)	− 0.001	− 0.001	< 0.001	< 0.001
ba-PWV (max)	0.0003	0.002	0.004	< 0.001
Global longitudinal strain				
ABI (min)	− 2.56	− 3.53	− 1.60	< 0.001
fa-PWV (max)	0.001	0.001	0.001	< 0.001
ba-PWV (max)	0.002	0.001	0.002	< 0.001
Global circumferential strain				
ABI (min)	− 2.04	− 3.66	− 0.42	0.014
fa-PWV (max)	0.001	< 0.001	0.001	0.076
ba-PWV (max)	< 0.001	< 0.001	0.001	0.111

ABI (min), minimal ankle-brachial index; fa-PWV (max), maximum femoral-ankle pulse wave velocity; ba-PWV (max), maximum brachial-ankle pulse wave velocity.

**Figure 2:**
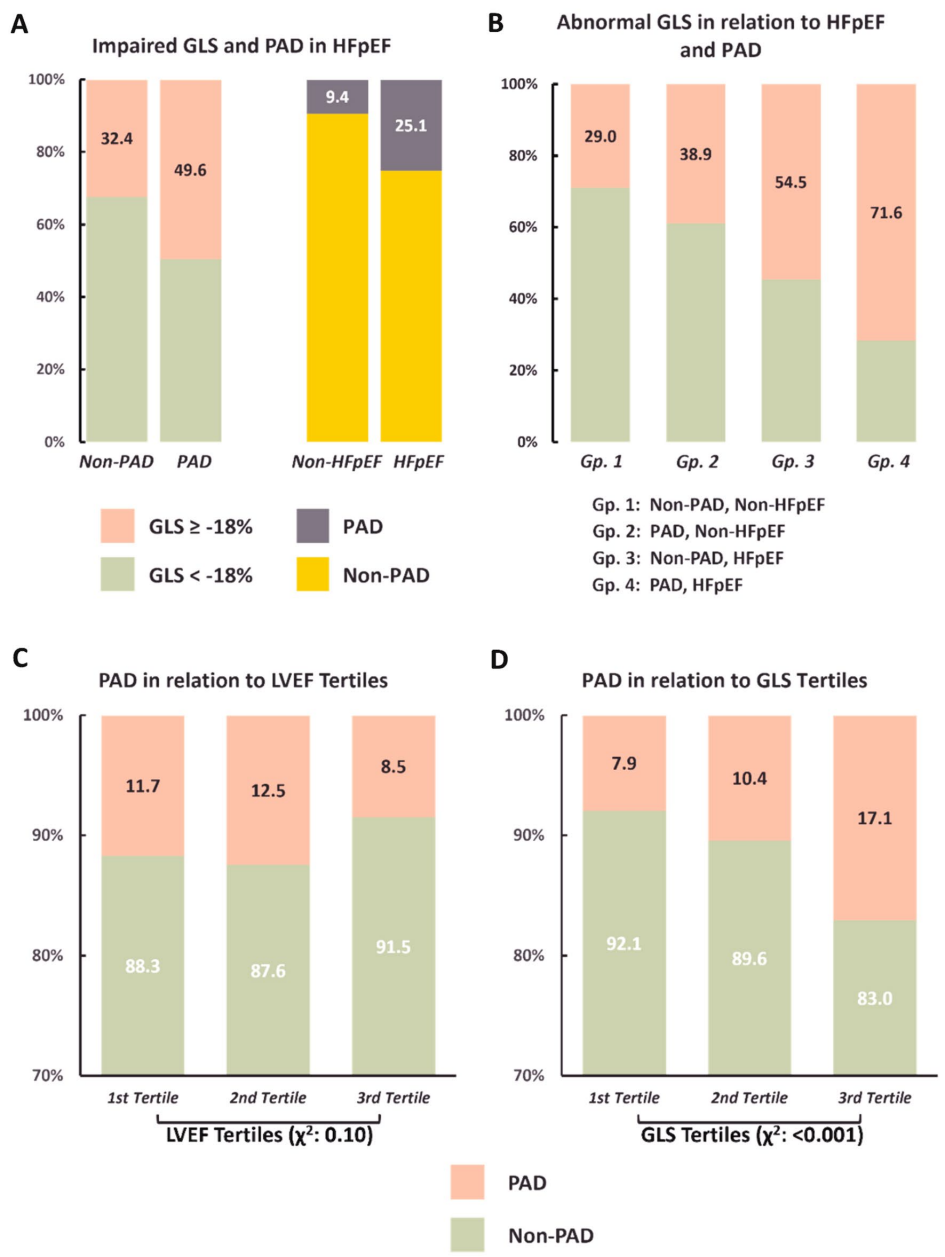
Associations of PAD with HFpEF and GLS. (**A**) Associations of prevalent high risks of developing PAD with HFpEF and impaired GLS (≥ − 18%). (**B**) Associations of impaired GLS of four strata based on the presence of high risks of developing PAD and HFpEF. (**C**, **D**) Associations of prevalent high risks of developing PAD with LVEF and GLS tertiles. Prevalent high risks of developing PAD was significantly increased across decreasing GLS rather than LVEF tertiles. PAD, peripheral artery disease; HEpEF, heart failure with preserved ejection fraction; GLS, global longitudinal strain; LVEF, left ventricular ejection fraction.

**Figure 3:**
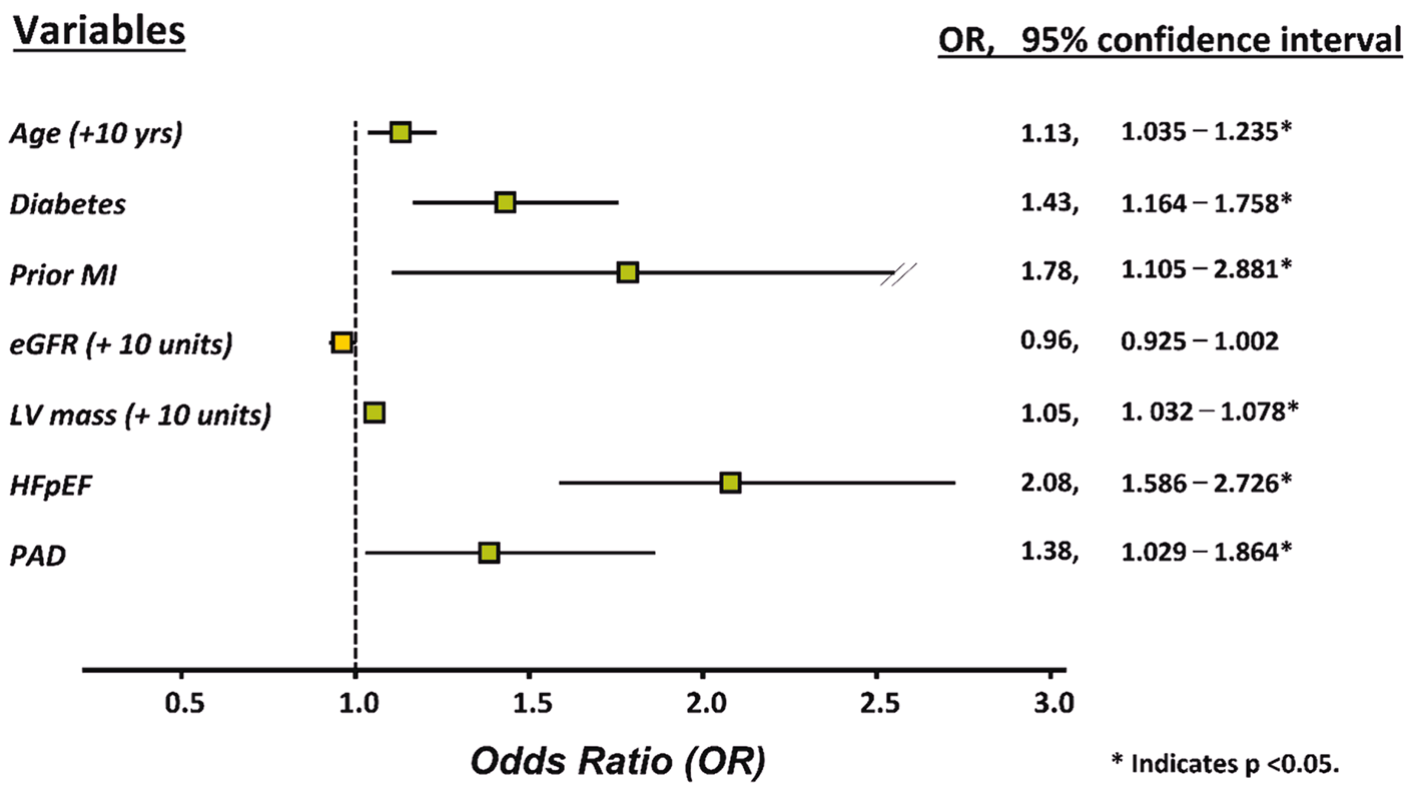
Forest plot for predictors of impaired GLS (≥ − 18%). Backward stepwise logistic regression analysis showing independent predictors of impaired GLS. Both the presence of high risks of developing PAD and HFpEF remained statistically significant associated with impaired GLS in model. Green, statistically significant; yellow, statistically non-significant. GLS, global longitudinal strain; MI, myocardial infarction; eGFR, estimated glomerular filtration rate; LV mass, left ventricle mass; HEpEF, heart failure with preserved ejection fraction; PAD, peripheral artery disease.

Figure 2 presents the associations of high risk of developing PAD, HFpEF, GLS and LVEF. High risk of developing PAD had more prevalence in HFpEF (25.1% vs. 9.4%) and more impaired GLS (49% vs. 32%, both $\chi^{2}$ p < 0.05) compared to low risk of developing PAD counterpart (Fig. 2A). Figure 2B showed the associations of impaired GLS of four groups based on high risk of developing PAD and HFpEF and showed impaired GLS is association with high risk of developing PAD and HFpEF (p for non-parametric linear trend: < 0.001). Further categorize to three tertiles based on LVEF and GLS. High risk of developing PAD is associated with LVEF (Fig. 2C) and impaired GLS (Fig. 2D). Figure 3 showed the independent predictors for impaired preclinical longitudinal systolic function defined as GLS ≥ − 18%. Both the presence of high risk of developing PAD (OR, 1.38; 95% Cl, 1.03–1.86) and HFpEF (OR, 2.08; 95% Cl, 1.59–2.73) were independently associated with impaired GLS by backward stepwise logistic regression analysis.

### The association of PAD and clinical endpoints

**Figure 4:**
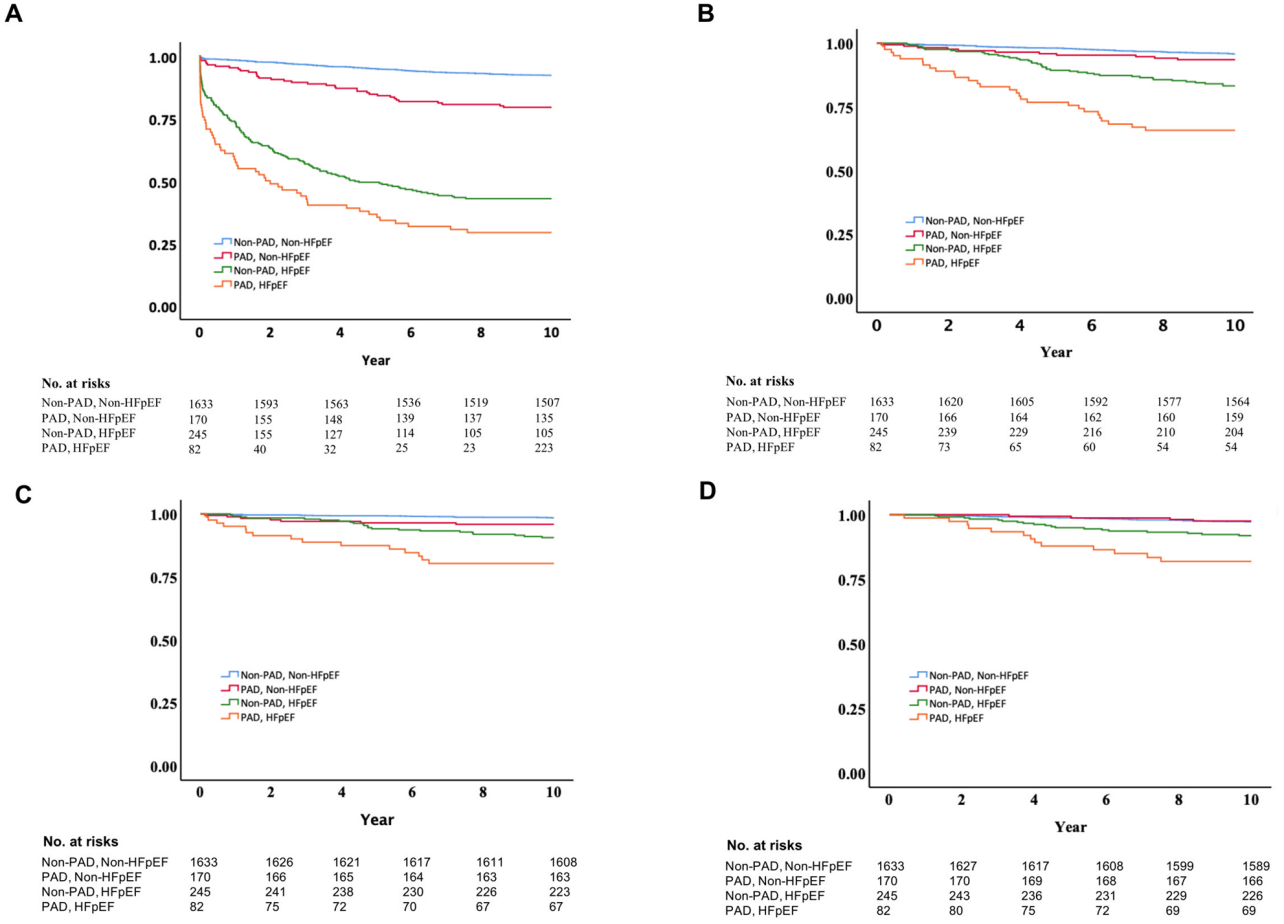
Kaplan–Meier plot of hospitalization for HF and death. Kaplan–Meier survival curves showing (**A**) hospitalization for HF and (**B**) all cause death from any cause according to PAD/HFpEF strata in the present study. HF, heart failure; PAD, peripheral artery disease.

The unadjusted cumulative incidence estimates for hospitalization for HF, all-cause mortality, CV mortality and non-CV mortality are shown in Fig. 4A–D respectively. Compared with Non-PAD/Non-HFpEF reference group, the unadjusted cumulative incidence estimates for hospitalization for HF, all-cause mortality, CV mortality and non-CV mortality were higher among patients with both high risk of developing PAD and HFpEF.

In the multivariate models adjusted for demographics (age, sex), comorbidities (current smoking, hypertension, CAD, diabetes mellitus, atrial fibrillation, hyperlipidemia), eGFR, and LV mass index, several significant findings emerged.

Patients with high risk of developing PAD without HFpEF (as PAD/Non-HFpEF group) had a higher risk of hospitalization for HF (HR, 1.71; 95% CI, 1.15–2.55) during the follow-up period. However, they did not exhibit an independent risk of all-cause mortality (HR, 0.67; 95% CI, 0.24–0.98), CV mortality (HR, 0.98; 95% CI, 0.40–2.39), or non-CV mortality (HR, 0.43; 95% CI, 0.15–1.21) when compared to the Non-PAD/Non-HFpEF reference group (Table 4 and Supplemental Tables 3–6). These results were observed after multivariate adjustments. The presence of high risk of developing PAD did not modify the risk of HF hospitalization among HFpEF patients in the whole study population in fully adjusted model (P_interaction_: 0.066).

**Table 4:** The associations among PAD, HFpEF and outcomes.

Outcome	Event No.	Unadjusted	Model 1	Model 2	Model 3
	N (%)	HR (95% CI)	HR (95% CI)	HR (95% CI)	HR (95% CI)
HF admission					
Non-PAD, Non-HFpEF	126 (7.8%)	*Ref.*	*Ref.*	*Ref.*	*Ref.*
PAD, Non-HFpEF	35 (22.9%)	2.86 (1.97–4.16)	2.19 (1.50–3.21)	2.21 (1.51–3.23)	1.71 (1.15–2.55)
Non-PAD, HFpEF	140 (57.1%)	11.16 (8.76–14.22)	8.85 (6.87–11.39)	9.18 (7.07–11.92)	6.09 (4.58–8.10)
PAD, HFpEF	59 (70.20%)	17.72 (12.98–24.19)	12.38 (8.83–17.36)	12.65 (9.00–17.78)	6.51 (4.43–9.55)
All cause Death					
Non-PAD, Non-HFpEF	69 (4.2%)	*Ref.*	*Ref.*	*Ref.*	*Ref.*
PAD, Non-HFpEF	11 (6.4%)	1.56(0.83–2.95)	0.83 (0.44–1.59)	0.83 (0.44–1.59)	0.67 (0.24–0.98)
Non-PAD, HFpEF	41 (16.7%)	4.23(2.87–6.22)	2.68 (1.81–3.98)	2.68 (1.80–4.00)	1.71 (1.15–2.73)
PAD, HFpEF	28 (34.1%)	10.08(6.49–15.64)	3.90 (2.41–6.31)	3.90 (2.41–6.33)	2.01 (1.17–3.38)
CV death					
Non-PAD, Non-HFpEF	25 (1.5%)	*Ref.*	*Ref.*	*Ref.*	*Ref.*
PAD, Non-HFpEF	7 (4.1%)	2.74(1.18–6.33)	1.33 (0.56–3.13)	1.34 (0.57–3.15)	0.98 (0.40–2.39))
Non-PAD, HFpEF	22 (9.0%)	6.22(3.51–11.04)	3.80 (2.12–6.81)	3.89 (2.16–7.01)	2.34 (1.18–4.66)
PAD, HFpEF	15 (18.3%)	14.62(7.70–27.75)	5.14 (2.55–10.34)	5.22 (2.59–10.55)	2.44 (1.08–5.51)
Non-CV death					
Non-PAD, Non-HFpEF	44 (2.7%)	*Ref.*	*Ref.*	*Ref.*	*Ref.*
PAD, Non-HFpEF	4 (2.4%)	0.89 (0.32–2.48)	0.51 (0.18–1.45)	0.51 (0.18–1.44)	0.43 (0.15–1.21)
Non-PAD, HFpEF	19 (7.8%)	3.09 (1.80–5.29)	2.02 (1.17–3.51)	1.98 (1.13–3.45)	1.38 (0.73–2.60)
PAD, HFpEF	13 (15.9%)	7.46 (4.18–13.86)	3.13 (1.59–6.14)	3.08 (1.57–6.07)	1.78 (0.82–3.84)

Group 1: Non-PAD, Non-HFpEF, Group 2: PAD, Non-HFpEF, Group 3: Non-PAD, HFpEF, Group 4: PAD, HFpEF.

Model 1: adjusted for age and sex; Model 2: adjusted for age, sex, BMI; Model 3: adjusted for age, sex, hypertension, diabetes, coronary artery disease, atrial fibrillation, hyperlipidemia, smoking status, eGFR and LV mass index.

Conversely, patients with low risk of developing PAD but with HFpEF (as Non-PAD/HFpEF group) displayed an increased risk of hospitalization for HF (HR, 6.09; 95% CI, 4.58–8.10), all-cause mortality (HR, 1.71; 95% CI, 1.15–2.73), CV mortality (HR, 2.34; 95% CI, 1.18–4.66), and non-CV mortality (HR, 1.38; 95% CI, 0.73–2.60) when compared to the Non-PAD/Non-HFpEF reference group (Table 4 and Supplemental Table 3–6). Patients with both high risk of developing PAD and HFpEF (as PAD/HFpEF group) displayed the highest risks of hospitalization for HF (HR, 6.51; 95% CI, 4.43–9.55), all-cause mortality (HR, 2.01; 95% CI, 1.17–3.38), CV mortality (HR, 2.44; 95% CI, 1.08–5.51), and non-CV mortality (HR, 1.78; 95% CI, 0.82–3.84) when compared to the Non-PAD/Non-HFpEF reference group (Table 4 and Supplemental Tables 3–6). These elevated risks were observed after multivariate adjustments. The presence of high risk of developing PAD further increased the risk of all-cause mortality among HFpEF patients in the whole study population in fully adjusted model (P_interaction_: 0.173).

## Discussion

Among a total of 2130 at-risk outpatients manifesting preserved LVEF in this retrospective observational study, we observed the following: (1) patients at a high risk of developing PAD were more prevalent in HFpEF population, and it was independently associated with more impaired longitudinal systolic function compared to those without HFpEF; (2) Compared with those at low risk of developing PAD and without HFpEF, patients with high risk of developing PAD and HFpEF had worst cardiac profiles (including longitudinal systolic function) and a highest risk of hospitalization for HF, all-cause mortality, CV mortality and non-CV mortality; (3) After adjustments, patients with high risk of developing and HFpEF were associated with a six-fold higher hazard of HF hospitalization, two-fold higher hazard of all-cause mortality and CV mortality when compared to patients with low risk of developing PAD and without HFpEF.

Previous investigations on the association of PAD with HFpEF and short-term follow-up from clinical trials have been inconsistent. In addition, not all the patients in these studies underwent comprehensive echocardiography. Over a 4-year follow-up period, a propensity score matching analysis among chronic HFpEF patients enrolled in the BEST (Beta-Blocker Evaluation of Survival Trial) reported no difference in ADHF hospitalization (HR, 1.05) and heart mortality (HR, 1.40) among clinically diagnosed PAD patients versus non-PAD counterpart^9^. The TOPCAT (Treatment of Preserved Cardiac Function Heart Failure With an Aldosterone Antagonist) trial, which mainly enrolled HFpEF patients, reported a significant increase of all-cause mortality (HR, 1.56) and non-significant increase in HF hospitalization during 3.4 years follow-up for PAD patients versus those without PAD (HR, 1.29)^11^. PAD is associated with a high burden of clinical comorbid conditions^12,13^. Recently, a patient-level meta-analysis of DAPA-HF and DELIVER showed a significant increase of HF hospitalization (HR, 1.24), all cause death (HR, 1.25) and CV death (HR, 1.22)^14^.

In our study, patients with high risk of developing PAD presented with a greater comorbidity burden compared to those with low risk of developing PAD. Individuals with high risk of developing PAD are associated with more impaired preclinical systolic function in the context of worsened GLS, along with more deteriorated diastolic function presenting lower LV myocardial relaxation e’ and higher LV filling pressure E/e′. More unfavorable vascular arterial indices were associated with more impaired cardiac diastolic and preclinical systolic functions (LV strains) in present study. Furthermore, after adjusting for comorbidities, patients with high risk of developing PAD remained strongly associated with hospitalization for HF as well as all-cause mortality, CV mortality and non-CV mortality. A previous study showed that the prevalence of LV diastolic dysfunction was higher in patients with PAD than in without PAD^15^, which is consistent with our study. Recently, ABI has shown to be associated with arterial stiffness via pressure wave reflection in middle-aged Japanese and Western populations free from overt clinical PAD^16–18^. One potential explanation for the association observed among PAD, impaired preclinical cardiac dysfunction and HF hospitalization may come from ED or altered arterial function, such as increased arterial stiffness. While arterial stiffness can co-exist with senescence process, ED per se is a unique pathological process that starts at the level of the endothelium, a dynamic, functionally complex organ involved in the regulation of several important biological mechanisms, including maintenance of vascular tone and permeability, inflammatory responses, immunity, and angiogenesis^19^. ED plays a central role in the development of atherosclerotic disorder and microvascular dysfunction, and it has been proposed that several cardiovascular risks (e.g., aging, obesity, diabetes, hyperlipidemia, or chronic kidney disease) may impair myocardial microvascular circulation even in the absence of evidenced or established of cardiovascular disease that may further impact myocardial erfusion and myocardial performance^8,20–23^. The shared common pathological link among excessive CV risks, vascular stiffness in PAD and HFpEF has been extensively investigated in recent decades^4,15,24,25^.

In recent years, there has been a notable increase in the utilization of sodium-glucose cotransporter 2 inhibitors (SGLT2-I), attributed to their established benefits in patients with cardiovascular disease and PAD^26–28^. The SGLT2-I AMI PROTECT Registry, for instance, revealed that the use of SGLT2-I independently predicted a reduction in heart failure hospitalization (HR = 0.46; 95%CI: 0.21–0.98; P = 0.041)^28^.

Both preclinical and clinical investigations have suggested that SGLT2-I exert a positive influence on endothelial and microvascular function through a combination of mechanisms, contributing to their favorable cardiovascular effects^29^.

Specifically, empagliflozin, a member of the SGLT2-I class, diminishes frailty in individuals with diabetes and hypertension. This effect is likely achieved by mitigating the generation of reactive oxygen species in endothelial cells^30^.

Moreover, a systematic review and meta-analysis aimed at assessing the impact of SGLT2-I on endothelial function and arteriosclerosis in diabetic patients demonstrated that SGLT-2 inhibitors exhibit superiority over other antidiabetic agents in enhancing arterial endothelial function. This underscores the potential of SGLT2-I as a promising therapeutic option with broader cardiovascular benefits for individuals with diabetes^31^.

### Limitation

The current study has several limitations. Firstly, it is important to note that our current findings were based on a retrospective study design. The extent to which our current research findings can be applied to broader community-based populations has not been investigated. The generalizability of our study primarily stems from data collected from outpatient settings. However, the applicability and relevance of these findings to populations within a community-based context have not been thoroughly explored. Furthermore, the vascular stiffness measures used in the current study are noninvasive and evidence of direct measure on ED cannot be obtained, and the data were restricted to a single center. Indirect measures of arterial stiffness using PWV and ABI are widely used as screening tools for peripheral artery disease have been widely used in clinical settings. Third, it is possible that the impact of PAD on HFpEF outcomes is greater than reported in this analysis, given that a majority of PAD patients are asymptomatic. However, due to a lack of specific data, we are unable to determine the number (and percentage) of asymptomatic patients and the types of symptoms reported by others.

## Conclusion

In summary, patients with high risk of developing PAD were more prevalent in HFpEF population, associated with more impaired preclinical systolic function and had a greater comorbidities burden. The association among PAD, impaired preclinical cardiac dysfunction and HF hospitalization may come from ED and arterial stiffness. Patients with both high risk of developing PAD and HFpEF also had a higher risk of hospitalization for HF, all-cause mortality, CV mortality and non-CV mortality compared with those with low risk of developing PAD and without HFpEF. Practitioners should be aware of the high risk of adverse outcomes associated with high risk of developing PAD in HFpEF and needed to develop strategies to prevent hospitalization for HF and all-cause mortality in this high-risk group patients.

### Supplementary Information

Supplementary Tables.

Supplementary Figure 1.

## Data Availability

The datasets generated and analysed during the current study are not publicly because of the sensitive nature of the data collected, but are available from the corresponding author on reasonable request.

## Abbreviations

ABI: Ankle-brachial index
BMI: Body mass index
CAD: Coronary artery disease
CV: Cardiovascular
ED: Endothelial dysfunction
E/e′: Mitral Doppler early velocity/mitral annular early velocity
eGFR: Estimated glomerular filtration rate
GLS/GCS: Global longitudinal/circumferential strain
HFpEF: Heart failure with reduced ejection fraction
PAD: Peripheral arterial disease
PWV: Pulse wave velocity
