# Supplementary material for: Preclinical systolic dysfunction relating to ankle-brachial index among high-risk PAD population with preserved left ventricular ejection fraction
Source: Sci Rep. 2024 Mar 14;14:6145. doi: 10.1038/s41598-024-52375-y (PMC10937714; doi:10.1038/s41598-024-52375-y)
Supplement: Supplementary file 1 — Supplementary Tables. [file 41598_2024_52375_MOESM1_ESM.docx]

| Supplemental Materials   \| **Supplemental Table 1. Baseline Characteristics** \| \| \| \| \| \| --- \| --- \| --- \| --- \| --- \| \|  \| **All**  **(n=2,130)** \| **Non-PAD**  **(n=1,878)** \| **PAD**  **(n=252)** \| **P value** \| \| **Biochemical data** \| \| \| \| \| \| **AC glucose(mg/dL)** \| 115.94±37.59 \| 114.59±35.90 \| 126.00±47.24 \| <0.001 \| \| **HbA1c (%)** \| 6.51±183 \| 6.47±1.87 \| 6.81±1.50 \| <0.001 \| \| **Total cholesterol (mg/dL)** \| 195.29±42.11 \| 195.94±41.80 \| 190.43±44.16 \| 0.046 \| \| **LDL (mg/dL)** \| 117.41±35.17 \| 118.30±34.92 \| 110.63±36.35 \| 0.001 \| \| **eGFR (MDRD),** (**mL/min/1.73 m^2^)** \| 80.41±28.11 \| 82.70±26.92 \| 63.27±30.90 \| <0.001 \| \| **Hs-CRP (mg/dL)** \| 0.37±0.88 \| 0.31±0.65 \| 0.90±1.86 \| <0.001 \| \| **BNP (pg/mL)** \| 87.25±287.76 \| 67.47±226.84 \| 226.85±531.87 \| <0.001 \| \| LDL: low density cholesterol; eGFR: estimated glomerular filtration rate; Hs-CRP: high-sensitivity C-reactive protein; BNP: B-type natriuretic peptide \| \| \| \| \|   Supplemental Table 2.  Univariate and Multivariate Cox Regression Analysis of Hospitalization for heart failure | | | | | | |
| --- | --- | --- | --- | --- | --- | --- | --- | --- | --- | --- | --- | --- | --- | --- | --- | --- | --- | --- | --- | --- | --- | --- | --- | --- | --- | --- | --- | --- | --- | --- | --- | --- | --- | --- | --- | --- | --- | --- | --- | --- | --- | --- | --- | --- | --- | --- | --- | --- | --- | --- | --- | --- | --- | --- | --- | --- | --- | --- | --- | --- | --- |
| **Variables** | **Univariate Analysis** | | | **Multivariate Analysis** | | |
|  | **HR** | **95%Cl** | ***p value*** | **HR** | **95%Cl** | ***p value*** |
| Non-PAD, Non-HFpEF |  | Ref. |  |  | Ref. |  |
| PAD, Non-HFpEF | 2.86 | 1.97-4.16 | <0.001 | 1.71 | 1.15-2.55 | 0.009 |
| Non-PAD, HFpEF | 11.16 | 8.76-14.22 | <0.001 | 6.09 | 4.58-8.10 | <0.001 |
| PAD, HFpEF | 17.72 | 12.98-24.19 | <0.001 | 6.51 | 4.43-9.55 | <0.001 |
| Age | 1.06 | 1.05-1.07 | <0.001 | 1.02 | 1.01-1.03 | <0.001 |
| Sex |  |  |  |  |  |  |
| female |  | Ref. |  |  | Ref. |  |
| male | 0.86 | 0.70-1.05 | 0.042 | 1.25 | 0.97-1.62 | 0.082 |
| Waist | 1.03 | 1.02-1.04 | <0.001 |  |  |  |
| BMI | 1.01 | 0.99-1.03 | 0.377 |  |  |  |
| Smoke | 1.12 | 0.88-1.42 | 0.348 | 1.15 | 0.87-1.52 | 0.330 |
| Hypertension | 0.80 | 0.63-1.01 | 0.060 | 0.75 | 0.59-0.96 | 0.022 |
| Diabetes mullites | 2.95 | 2.39-3.63 | <0.001 | 1.51 | 1.20-1.90 | <0.001 |
| Coronary artery disease | 3.44 | 2.79-4.26 | <0.001 | 1.51 | 1.18-1.92 | 0.001 |
| Atrial fibrillation | 4.09 | 3.05-5.49 | <0.001 | 0.78 | 0.56-1.08 | 0.135 |
| Hyperlipidemia | 1.11 | 0.90-1.37 | 0.327 | 0.91 | 0.72-1.14 | 0.397 |
| Systolic blood pressure | 1.01 | 1.01-1.01 | <0.001 |  |  |  |
| eGFR | 0.97 | 0.97-0.97 | <0.001 | 0.98 | 0.98-0.99 | <0.001 |
| LV mass index | 1.02 | 1.02-1.02 | <0.001 | 1.01 | 1.00-1.01 | 0.001 |
| Relative mass index | 22.61 | 5.58-91.59 | <0.001 |  |  |  |
| LA diameter | 1.05 | 1.04-1.07 | <0.001 |  |  |  |
| LVEF | 0.96 | 0.94-0.98 | <0.001 |  |  |  |
| IVRT | 1.00 | 1.00-1.01 | 0.123 |  |  |  |
| DT | 1.00 | 1.00-1.00 | 0.001 |  |  |  |
| Septal e’ | 0.77 | 0.72-0.81 | <0.001 |  |  |  |
| Lateral e’ | 0.84 | 0.81-0.88 | <0.001 |  |  |  |
| E’/e’ | 1.07 | 1.06-1.08 | <0.001 |  |  |  |
| GLS | 1.21 | 1.17-1.24 | <0.001 |  |  |  |
| GCS | 1.05 | 1.03-1.08 | <0.001 |  |  |  |
| ABI (min) | 0.06 | 0.03-0.11 | <0.001 |  |  |  |
| fa-PWV (max) | 1.00 | 1.00-1.00 | <0.001 |  |  |  |
| ba-PWV (max) | 1.00 | 1.00-1.00 | <0.001 |  |  |  |
| Abbreviations  BMI: body mass index; eGFR: estimated glomerular filtration rate; LV mass index: left ventricle mass index; LA diameter: left atrial diameter; LVEF: left ventricular ejection fraction; IVRT: isovolumetric relaxation time; DT: Deceleration time; GLS: global longitudinal strain; GCS: global circumferential strain; ABI (min): minimal ankle-brachial index; ba-PWV (max): femoral-ankle pulse wave velocity; ba-PWV: brachial-ankle pulse wave velocity. | | | | | | |

| Supplemental Table 3.  Univariate and Multivariate Cox Regression Analysis of All Cause Death | | | | | | |
| --- | --- | --- | --- | --- | --- | --- |
| **Variables** | **Univariate Analysis** | | | **Multivariate Analysis** | | |
|  | HR | 95%Cl | *p value* | HR | 95%Cl | *p value* |
| Non-PAD, Non-HFpEF |  | Ref. |  |  | Ref. |  |
| PAD, Non-HFpEF | 1.56 | 0.83-2.95 | 0.170 | 0.67 | 0.24-0.98 | 0.229 |
| Non-PAD, HFpEF | 4.23 | 2.87-6.22 | <0.001 | 1.71 | 1.15-2.73 | 0.024 |
| PAD, HFpEF | 10.08 | 6.49-15.64 | <0.001 | 2.01 | 1.17-3.38 | 0.014 |
| Age | 1.09 | 1.08-1.11 | <0.001 | 1.08 | 1.05-1.09 | <0.001 |
| Sex |  |  |  |  |  |  |
| female | Ref. | | | Ref. | | |
| male | 1.35 | 0.98-1.87 | 0.068 | 1.78 | 1.06-2.18 | 0.003 |
| Waist | 1.02 | 1.01-1.04 | 0.004 |  |  |  |
| BMI | 0.98 | 0.94-1.02 | 0.284 |  |  |  |
| Smoke | 1.00 | 0.69-1.46 | 0.989 | 0.93 | 0.63-1.45 | 0.736 |
| Hypertension | 0.83 | 0.58-1.19 | 0.307 | 0.84 | 0.61-1.27 | 0.387 |
| Diabetes mullites | 2.89 | 2.09-4.00 | <0.001 | 2.03 | 1.36-2.63 | <0.001 |
| Coronary artery disease | 2.27 | 1.61-3.20 | <0.001 | 1.36 | 0.89-1.88 | 0.128 |
| Atrial fibrillation | 4.05 | 2.61-6.26 | <0.001 | 0.96 | 0.61-1.60 | 0.876 |
| Hyperlipidemia | 0.79 | 0.57-1.08 | 0.141 | 0.73 | 0.56-1.08 | 0.083 |
| Systolic blood pressure | 1.00 | 0.99-1.01 | 0.792 |  |  |  |
| eGFR | 0.97 | 0.96-0.97 | <0.001 | 0.99 | 0.98-0.99 | <0.001 |
| LV mass index | 1.01 | 1.00-1.02 | 0.020 | 0.99 | 0.98-1.00 | 0.029 |
| Relative mass index | 10.69 | 1.19-95.68 | 0.034 |  |  |  |
| LA diameter | 1.03 | 1.01-1.06 | 0.006 |  |  |  |
| LVEF | 0.96 | 0.94-0.98 | 0.001 |  |  |  |
| IVRT | 1.01 | 1.00-1.01 | 0.023 |  |  |  |
| DT | 1.00 | 1.00-1.01 | <0.001 |  |  |  |
| Septal e’ | 0.77 | 0.70-0.84 | <0.001 |  |  |  |
| Lateral e’ | 0.83 | 0.77-0.89 | <0.001 |  |  |  |
| E’/e’ | 1.07 | 1.05-1.08 | <0.001 |  |  |  |
| GLS | 1.21 | 1.16-1.27 | <0.001 |  |  |  |
| GCS | 1.05 | 1.01-1.09 | 0.006 |  |  |  |
| ABI (min) | 0.12 | 0.05-0.30 | <0.001 |  |  |  |
| fa-PWV (max) | 1.00 | 1.00-1.00 | 0.095 |  |  |  |
| ba-PWV (max) | 1.00 | 1.00-1.00 | <0.001 |  |  |  |
| Abbreviations  BMI: body mass index; eGFR: estimated glomerular filtration rate; LV mass index: left ventricle mass index; LA diameter: left atrial diameter; LVEF: left ventricular ejection fraction; IVRT: isovolumetric relaxation time; DT: Deceleration time; GLS: global longitudinal strain; GCS: global circumferential strain; ABI (min): minimal ankle-brachial index; ba-PWV (max): femoral-ankle pulse wave velocity; ba-PWV: brachial-ankle pulse wave velocity | | | | | | |

| Supplemental Table 4.  Univariate and Multivariate Cox Regression Analysis of CV Death | | | | | | |
| --- | --- | --- | --- | --- | --- | --- |
| **Variables** | **Univariate Analysis** | | | **Multivariate Analysis** | | |
|  | HR | 95%Cl | *p value* | HR | 95%Cl | *p value* |
| Non-PAD, Non-HFpEF |  | Ref. |  |  | Ref. |  |
| PAD, Non-HFpEF | 2.74 | 1.18-6.33 | 0.019 | 0.98 | 0.40-2.39 | 0.110 |
| Non-PAD, HFpEF | 6.22 | 3.51-11.04 | <0.001 | 2.34 | 1.18-4.66 | 0.326 |
| PAD, HFpEF | 14.62 | 7.70-27.75 | <0.001 | 2.44 | 1.08-5.51 | 0.032 |
| Age | 1.11 | 1.08-1.13 | <0.001 | 1.09 | 1.06-1.12 | <0.001 |
| Sex |  |  |  |  |  |  |
| female | Ref. | | | Ref. | | |
| male | 1.47 | 0.92-2.37 | 0.111 | 2.01 | 1.14-3.56 | 0.016 |
| Waist | 1.01 | 0.99-1.03 | 0.283 |  |  |  |
| BMI | 0.96 | 0.90-1.02 | 0.158 |  |  |  |
| Smoke | 1.07 | 0.62-1.85 | 0.814 | 1.02 | 0.54-1.92 | 0.947 |
| Hypertension | 0.70 | 0.42-1.16 | 0.165 | 0.70 | 0.40-1.23 | 0.219 |
| Diabetes mullites | 5.03 | 2.99-8.45 | <0.001 | 3.52 | 2.00-6.18 | <0.001 |
| Coronary artery disease | 2.09 | 1.25-3.48 | 0.005 | 1.01 | 0.56-1.83 | 0.977 |
| Atrial fibrillation | 5.80 | 3.27-10.27 | <0.001 | 1.14 | 0.59-2.23 | 0.694 |
| Hyperlipidemia | 0.84 | 0.53-1.35 | 0.478 | 0.82 | 0.49-1.39 | 0.465 |
| Systolic blood pressure | 0.99 | 0.98-1.01 | 0.341 |  |  |  |
| eGFR | 0.97 | 0.96-0.97 | <0.001 | 0.99 | 0.98-1.00 | 0.023 |
| LV mass index | 1.02 | 1.01-1.02 | 0.002 | 1.00 | 0.99-1.01 | 0.436 |
| Relative mass index | 74.39 | 3.38-1634.87 | 0.006 |  |  |  |
| LA diameter | 1.04 | 1.01-1.08 | 0.024 |  |  |  |
| LVEF | 0.94 | 0.91-0.98 | 0.001 |  |  |  |
| IVRT | 1.00 | 0.99-1.01 | 0.635 |  |  |  |
| DT | 1.00 | 1.00-1.01 | 0.078 |  |  |  |
| Septal e’ | 0.64 | 0.55-0.74 | <0.001 |  |  |  |
| Lateral e’ | 0.75 | 0.68-0.84 | <0.001 |  |  |  |
| E’/e’ | 1.08 | 1.06-1.10 | <0.001 |  |  |  |
| GLS | 1.24 | 1.16-1.32 | <0.001 |  |  |  |
| GCS | 1.03 | 0.97-1.08 | 0.350 |  |  |  |
| ABI (min) | 0.06 | 0.02-0.19 | <0.001 |  |  |  |
| fa-PWV (max) | 1.00 | 1.00-1.00 | 0.260 |  |  |  |
| ba-PWV (max) | 1.00 | 1.00-1.00 | <0.001 |  |  |  |
| Abbreviations  CV: cardiovascular; BMI: body mass index; eGFR: estimated glomerular filtration rate; LV mass index: left ventricle mass index; LA diameter: left atrial diameter; LVEF: left ventricular ejection fraction; IVRT: isovolumetric relaxation time; DT: Deceleration time; GLS: global longitudinal strain; GCS: global circumferential strain; ABI (min): minimal ankle-brachial index; ba-PWV (max): femoral-ankle pulse wave velocity; ba-PWV: brachial-ankle pulse wave velocity; | | | | | | |

| Supplemental Table 5.  Univariate and Multivariate Cox Regression Analysis of Non-CV Death | | | | | | |
| --- | --- | --- | --- | --- | --- | --- |
| **Variables** | **Univariate Analysis** | | | **Multivariate Analysis** | | |
|  | HR | 95%Cl | *p value* | HR | 95%Cl | *p value* |
| Non-PAD, Non-HFpEF |  | Ref. |  |  | Ref. |  |
| PAD, Non-HFpEF | 0.89 | 0.32-2.48 | 0.825 | 0.43 | 0.15-1.21 | 0.110 |
| Non-PAD, HFpEF | 3.09 | 1.80-5.29 | <0.001 | 1.38 | 0.73-2.60 | 0.326 |
| PAD, HFpEF | 7.46 | 4.18-13.86 | <0.001 | 1.78 | 0.82-3.84 | 0.143 |
| Age | 1.08 | 1.06-1.10 | <0.001 | 1.07 | 1.04-1.09 | <0.001 |
| Sex |  |  |  |  |  |  |
| female | Ref. | | | Ref. | | |
| male | 1.25 | 0.81-1.95 | 0.311 | 1.64 | 0.98-2.74 | 0.059 |
| Waist | 1.03 | 1.01-1.05 | 0.001 |  |  |  |
| BMI | 1.00 | 0.95-1.05 | 0.864 |  |  |  |
| Smoke | 0.95 | 0.56-1.60 | 0.842 | 0.86 | 0.47-1.56 | 0.610 |
| Hypertension | 0.97 | 0.58-1.63 | 0.921 | 1.01 | 0.59-1.74 | 0.962 |
| Diabetes mullites | 1.87 | 1.21-2.90 | 0.006 | 1.34 | 0.83-2.16 | 0.227 |
| Coronary artery disease | 2.44 | 1.54-3.88 | <0.001 | 1.78 | 1.04-3.01 | 0.037 |
| Atrial fibrillation | 2.70 | 1.35-5.39 | 0.005 | 0.71 | 0.32-1.59 | 0.404 |
| Hyperlipidemia | 0.74 | 0.48-1.15 | 0.177 | 0.67 | 0.41-1.07 | 0.092 |
| Systolic blood pressure | 1.00 | 0.99-1.01 | 0.603 |  |  |  |
| eGFR | 0.97 | 0.96-0.98 | <0.001 | 0.98 | 0.97-0.99 | 0.001 |
| LV mass index | 1.00 | 0.99-1.01 | 0.724 | 0.99 | 0.98-1.00 | 0.020 |
| Relative mass index | 1.42 | 0.08-25.13 | 0.809 |  |  |  |
| LA diameter | 1.03 | 0.99-1.06 | 0.103 |  |  |  |
| LVEF | 0.98 | 0.95-1.01 | 0.178 |  |  |  |
| IVRT | 1.01 | 1.01-1.02 | <0.001 |  |  |  |
| DT | 1.01 | 1.00-1.01 | 0.001 |  |  |  |
| Septal e’ | 0.88 | 0.78-0.98 | 0.026 |  |  |  |
| Lateral e’ | 0.89 | 0.81-0.97 | 0.008 |  |  |  |
| E’/e’ | 1.06 | 1.03-1.08 | <0.001 |  |  |  |
| GLS | 1.19 | 1.12-1.27 | <0.001 |  |  |  |
| GCS | 1.07 | 1.02-1.12 | 0.005 |  |  |  |
| ABI (min) | 0.28 | 0.06-1.21 | 0.087 |  |  |  |
| fa-PWV (max) | 1.00 | 1.00-1.00 | 0.219 |  |  |  |
| ba-PWV (max) | 1.00 | 1.00-1.00 | <0.001 |  |  |  |
| Abbreviations  CV: cardiovascular; BMI: body mass index; eGFR: estimated glomerular filtration rate; LV mass index: left ventricle mass index; LA diameter: left atrial diameter; LVEF: left ventricular ejection fraction; IVRT: isovolumetric relaxation time; DT: Deceleration time; GLS: global longitudinal strain; GCS: global circumferential strain; ABI (min): minimal ankle-brachial index; ba-PWV (max): femoral-ankle pulse wave velocity; ba-PWV: brachial-ankle pulse wave velocity; | | | | | | |

**Supplemental Figure Legends**

**Supplemental Figure 1. Flow chart of classification of based on risks of developing PAD with or without HFpEF.**
