# Supplementary figures and images for: Preclinical systolic dysfunction relating to ankle-brachial index among high-risk PAD population with preserved left ventricular ejection fraction
Source: Sci Rep. 2024 Mar 14;14:6145. doi: 10.1038/s41598-024-52375-y (PMC10937714; doi:10.1038/s41598-024-52375-y)

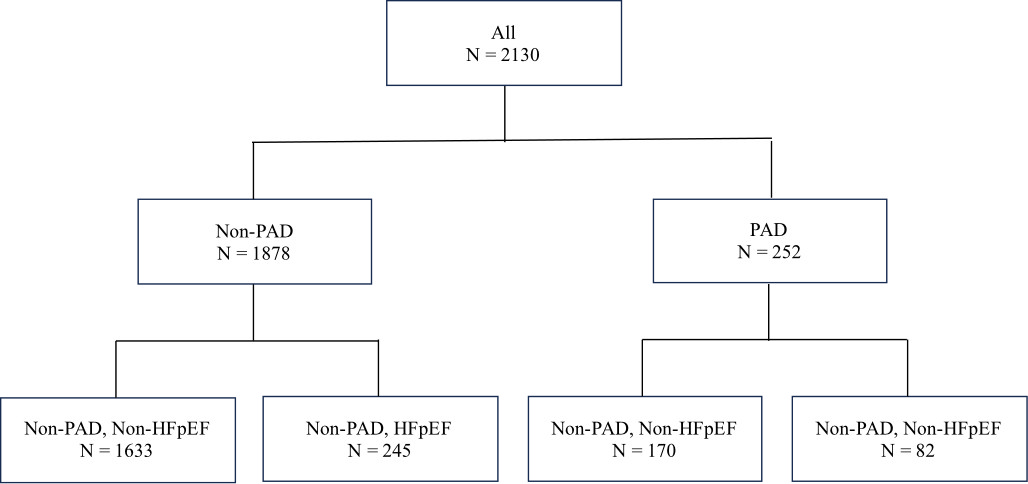

Supplement: Supplementary file 2 — Supplementary Figure 1. [file 41598_2024_52375_MOESM2_ESM.tif]
